# The CanMoRe trial – evaluating the effects of an exercise intervention after robotic-assisted radical cystectomy for urinary bladder cancer: the study protocol of a randomised controlled trial

**DOI:** 10.1186/s12885-020-07140-5

**Published:** 2020-08-26

**Authors:** Andrea Porserud, Patrik Karlsson, Elisabeth Rydwik, Markus Aly, Lars Henningsohn, Malin Nygren-Bonnier, Maria Hagströmer

**Affiliations:** 1 Department of Neurobiology, Care sciences and Society, Division of Physiotherapy, Karolinska Institutet, Stockholm, Sweden; 2 Allied Health Professionals Function, Medical unit Occupational Therapy and Physiotherapy, Karolinska University Hospital, Stockholm, Sweden; 3 Allied Health Professionals Function, Medical unit Ageing, Health and Function, Karolinska University Hospital, Stockholm, Sweden; 4 Department of Medical Epidemiology and Biostatistics, Karolinska Institutet, Stockholm, Sweden; 5 Patient Area Pelvic Cancer, Prostate Cancer Patient Flow, Karolinska University Hospital, Stockholm, Sweden; 6 Department of Molecular Medicine and Surgery, Karolinska Institutet, Stockholm, Sweden; 7 Department of Clinical Science, Intervention and Technology, CLINTEC, Division of Urology, Karolinska Institutet, Stockholm, Sweden; 8 Region Stockholm, Academic Primary Health Care Centre, Stockholm, Sweden

**Keywords:** Abdominal surgery, Behaviour, Bladder neoplasm, Complications, Exercise, Physical activity, Primary health care, Process evaluation

## Abstract

**Background:**

Patients who have undergone radical cystectomy for urinary bladder cancer are not sufficiently physically active and therefore may suffer complications leading to readmissions. A physical rehabilitation programme early postoperatively might prevent or at least alleviate these potential complications and improve physical function. The main aim of the CanMoRe trial is to evaluate the impact of a standardised and individually adapted exercise intervention in primary health care to improve physical function (primary outcome) and habitual physical activity, health-related quality of life, fatigue, psychological wellbeing and readmissions due to complications in patients undergoing robotic-assisted radical cystectomy for urinary bladder cancer.

**Methods:**

In total, 120 patients will be included and assigned to either intervention or control arm of the study. All patients will receive preoperative information on the importance of early mobilisation and during the hospital stay they will follow a standard protocol for enhanced mobilisation. The intervention group will be given a referral to a physiotherapist in primary health care close to their home. Within the third week after discharge, the intervention group will begin 12 weeks of biweekly exercise. The exercise programme includes aerobic and strengthening exercises. The control group will receive oral and written information about a home-based exercise programme.

Physical function will serve as the primary outcome and will be measured using the Six-minute walk test. Secondary outcomes are gait speed, handgrip strength, leg strength, habitual physical activity, health-related quality of life, fatigue, psychological wellbeing and readmissions due to complications. The measurements will be conducted at discharge (i.e. baseline), post-intervention and 1 year after surgery. To evaluate the effects of the intervention mixed or linear regression models according to the intention to treat procedure will be used.

**Discussion:**

This proposed randomised controlled trial has the potential to provide new knowledge within rehabilitation after radical cystectomy for urinary bladder cancer. The programme should be easy to apply to other patient groups undergoing abdominal surgery for cancer and has the potential to change the health care chain for these patients.

**Trial registration:**

ClinicalTrials.gov. Clinical trial registration number NCT03998579. First posted June 26, 2019.

## Background

The most common treatment for solid cancer tumours is surgery, often in combination with chemotherapy or radiotherapy, or both. Minimising postoperative complications is important in health care for the individual patient and in reducing health care costs for society. Early mobilisation at the ward and physical activity at home after discharge are important activities to reduce complications [1]. Common complications after abdominal surgery are postoperative pulmonary complications and venous thrombosis [2, 3], which generally are thought to be partially avoidable with early mobilisation.

After radical cystectomy for urinary bladder cancer, there is a high risk for postoperative complications. The complications could be directly related to the patients’ high age, to a high degree of comorbidity, or both [4]. The major risk factor for developing urinary bladder cancer is smoking [5]. Most of the patients are men and the median age of undergoing a radical cystectomy is 70 years [6, 7]. As much as 27% of patients are at severe nutritional risk before a radical cystectomy [8]. After robotic-assisted radical cystectomy (RARC) for urinary bladder cancer, 19–75% of the patients need to be readmitted to hospital after discharge because of complications [4, 9].

There is strong evidence that aerobic physical activity has a positive impact on health, survival and quality of life (QoL) [10]. Patients diagnosed with cancer should follow the general recommendations on physical activity and exercise for health [11, 12]. Yet, most patients are insufficiently active [13]. Consequently, with an increasing number of cancer survivors, the importance of supporting high physical function and QoL increases [14]. Research has shown that exercise has a positive effect on health-related QoL (HRQoL) in patients who have completed active cancer treatment [15]. Moreover, in patients living with or beyond a diagnosis of cancer, behavioural support methods (e.g., goal-setting and graded tasks) are important components in the exercise interventions with high adherence and positive physical outcomes [16].

In a recent study we evaluated the Activity Board® (Phystec, Sweden) as a method to enhance mobilisation and recovery after abdominal surgery for cancer. The Activity board is a tool based on techniques to support behaviour change [17, 18]. The evaluation showed that the Activity Board resulted in a higher level of mobilisation in the group with the Activity Board compared with the group who received standard treatment [19]. Although evidence for exercise after abdominal surgery is scarce [20], a few studies have evaluated exercise programmes for patients postoperatively at the hospital ward with promising results [21, 22].

Despite the lack of exercise interventions after surgery, it has been shown that functional performance after a radical cystectomy for urinary bladder cancer correlates to overall survival [23]. A large proportion of patients with urinary bladder cancer do not achieve the recommendations on physical activity and exercise [24]. It is also common that patients who undergo radical cystectomy have not performed physical exercise for a long time before surgery [24]. Finally, after surgery, patients report a low level of physical exercise [25]. Recently, two reviews have been published on physical and psychological interventions to improve health-related outcomes in this patient group [26, 27]. Both reviews include the same two postoperative exercise studies [28, 29]. One larger RCT showed that early physical exercise and enhanced mobilisation after radical cystectomy positively affected some domains of HRQoL [29]. In addition, in a pilot study we tested a model for physical rehabilitation after radical cystectomy [28]. The model consisted of 12 weeks of individually tailored exercise after discharge from the hospital. The exercise programme, conducted at the hospital, showed both short- and long-term effects on physical function and HRQoL.

Consequently, the few studies within the field raise several research questions for future exercise interventions in patients with urinary bladder cancer undergoing radical cystectomy. Current recommendations propose the following areas: supervised exercise after discharge, the optimal type of exercise, fidelity and adherence of the intervention, if short-term outcomes are sustained, clinical relevance, long-term outcomes and readmissions to hospital [26, 27, 30]. We also need to understand the kinds of support that are optimal, use behaviour change strategies and implement the intervention as a part of the patients’ clinical pathway through the healthcare system [26, 27, 31, 32].

In summary, patients who have been treated for urinary bladder cancer are not sufficiently physically active and suffer from readmissions to hospital due to complications. Therefore, there is a need for developing a physical rehabilitation programme to support patients who have a radical cystectomy in the early postoperative period. In this paper we present a study protocol for the CanMoRe trial: a physical rehabilitation programme after RARC for urinary bladder cancer.

## Methods/design

### Main objective

The main aim of the CanMoRe trial is to evaluate the impact of a standardised and individually adapted exercise intervention in primary health care (PHC) to improve physical function (primary outcome) and habitual physical activity, HRQoL, fatigue, psychological wellbeing and readmissions due to complications in patients undergoing RARC for urinary bladder cancer.

### Hypothesis

We hypothesise that the CanMoRe programme is more beneficial than home-based exercises in increasing physical function (primary outcome).

### Trial design

The CanMoRe trial is a randomised controlled trial (RCT) with a single-blinded design. The intervention group will receive a 12-week (1 h twice a week) standardised and individually adapted exercise intervention in PHC and behavioural support for daily physical activity. The control group will receive a home-based exercise programme as well as recommendations on daily physical activity based on general guidelines. We will follow the SPIRIT (Standard Protocol Items: Recommendations for Interventional Trials) 2013 statement and guidelines for reporting the study protocol. The clinical trial registration number for this trial is NCT03998579.

### Study setting

The study will be conducted in two settings: a university hospital and a PHC context in Region Stockholm.

### Recruitment and screening

**Fig. 1:**
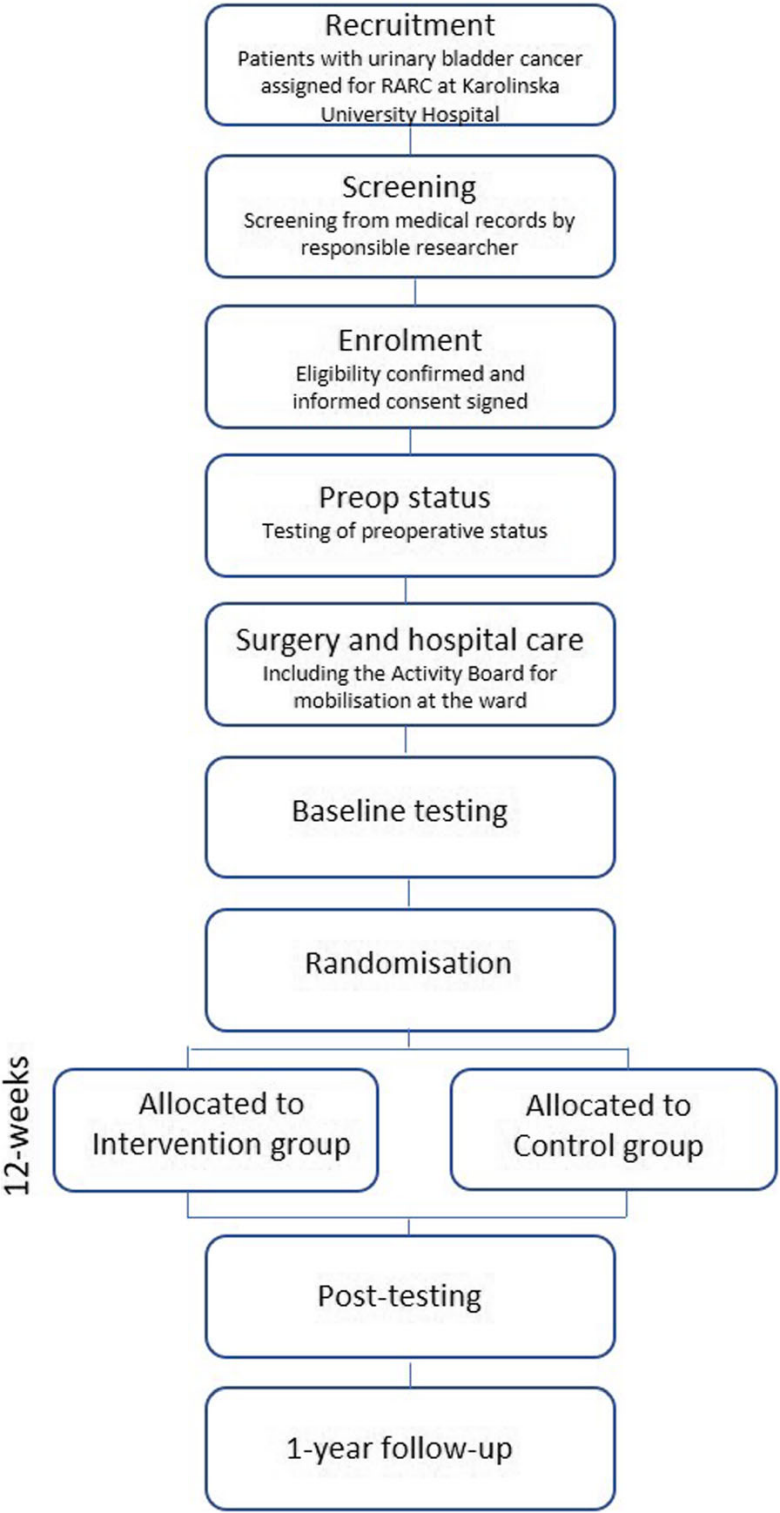
Patient flowchart

Participants will be recruited through Theme Cancer at the Karolinska University Hospital, Solna and screened for eligibility. Recruitment will be performed consecutively. Based on power analysis, 120 patients will be included. Potential participants will initially be screened for eligibility in medical records by the responsible researcher (RR) and given written information by a registered nurse at a preoperative meeting. After 2–3 days, the RR will phone the patient, provide oral information and then ask about participation. Informed consent will be signed before surgery. The RR keeps a protocol for enrolment. The patient flowchart is depicted in Fig. 1.

### Eligibility criteria

Inclusion criteria will be patients who are planned for a RARC for urinary bladder cancer. The patients should be able to talk and understand Swedish without an interpreter, be mobile with or without a walking aid and live in the Stockholm region.

#### Exclusion criteria

Patients with planned palliative surgery or cognitive impairment, identified by screening of medical records, will not be included.

### Randomisation – assignment of intervention

Patients who fulfil the criteria for inclusion will, after having given their oral and written consent, be randomised in the ALEA system, operated by the Clinical Trials Office (CTO) at the Centre for Clinical Cancer studies, Theme Cancer, Karolinska University Hospital, Solna, Sweden.

Randomisation will be conducted in blocks of 2–6 patients, stratified by sex and age (< 75, ≥75 years). A confirmation e-mail will be sent to the entering investigator and an enrolment log will be filed at the centre. The patient will receive the next consecutive code number in the trial and treatment arm according to the randomisation scheme.

### Logic model for the CanMoRe programme

It is recommended that programme design should be based on a theory to improve evidence synthesis [33]. The theoretical framework underpinning the CanMoRe programme is the Movement Continuum Theory (MCT) and the evidence-based CALO-RE taxonomy for behavioural change techniques. The MCT posits that an individual has three stages of movement capability: a maximum, a current and a preferred [34]. The CanMoRe programme identifies the patient’s current physical function (capability) and intervenes regarding the patient’s need for function. Several models and theories are supporting a behaviour change, which results in increased physical activity [16]. However, research has shown that it is most often not necesssary for a complete theory, but instead the different components in the theory that support the behaviour. To consider the patients need for support, the CALO-RE taxonomy for behavioural change techniques has been added [17]. The taxonomy is recommended to be used to improve the specification of interventions. Behavioural techniques proven effective to support behaviour change are goal-setting, graded tasks, self-monitoring, feedback and reward; all of these are used in the CanMoRe programme.

**Fig. 2:**
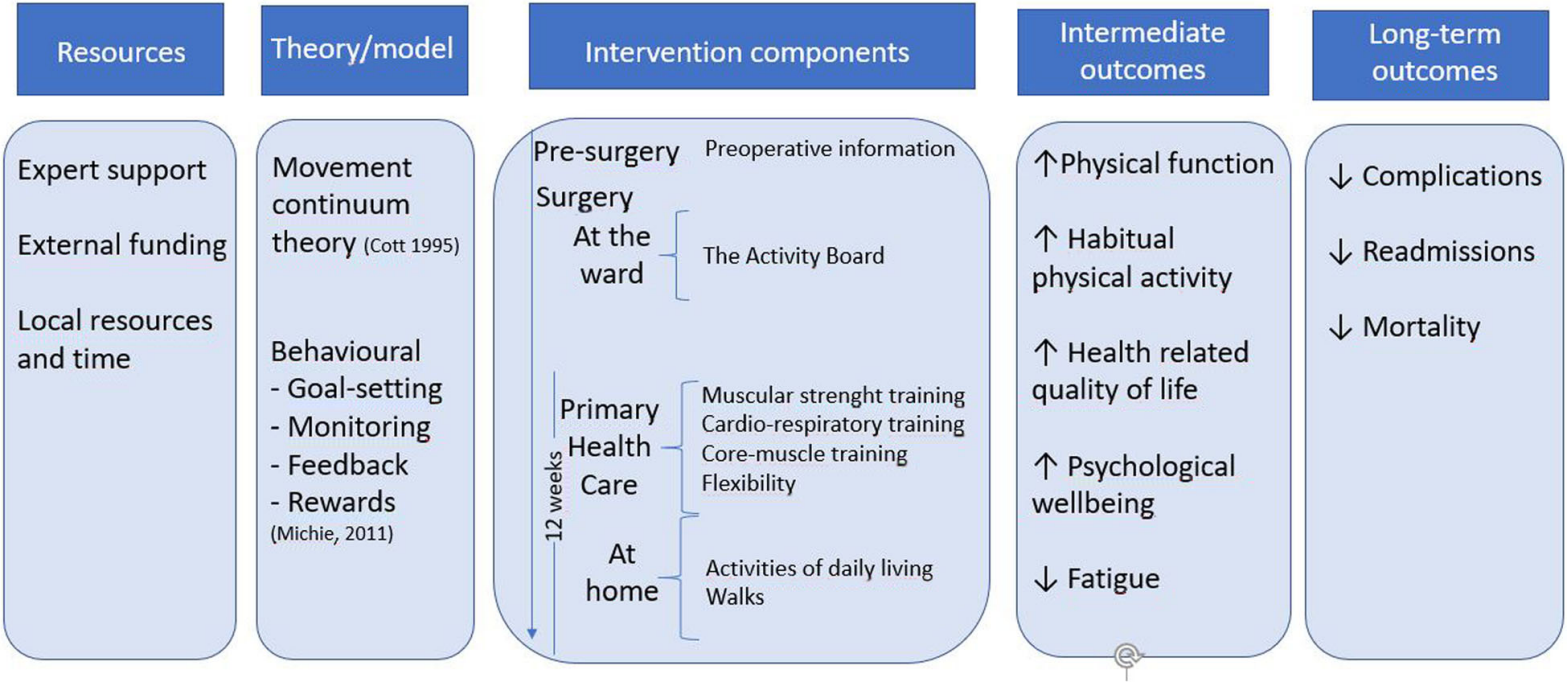
Logic model of the intervention

A conceptual framework visualising the inputs, theory, intervention components and its intermediate and possible long-term outcomes is depicted in a logic model (Fig. 2).

### Intervention

All patients receive preoperative information on the importance of early mobilisation and postoperative individual physiotherapy. During the hospital stay, the Activity Board is used for enhanced mobilisation. Before discharge from the hospital, the patients receive standardised information about avoiding the lifting of heavy objects and the importance of physical activity. The patients are then randomised to either the intervention or the control group.

#### Intervention group

Patients in the intervention group get a referral to a physiotherapist in PHC close to where they live. The patients can choose from 18 PHC settings spread throughout the Stockholm region. Within the third week after discharge, the patients begin 12 weeks of biweekly exercise. The patients pay for their primary care visits. Physiotherapists in the targeted primary care units receive a leaflet and a short education before starting comprising information about RARC, restrictions, potential adverse events, the trial process and the exercise programme. The physical exercise is individually targeted but based on international recommendations for persons with cancer disease. The exercise programme includes aerobic exercise aiming at moderate intensity (30 min/session) and strengthening exercises comprising endurance training with 2 × 15 repetitions (see Additional file 1). The programme is gradually increased based on the patient’s capability. The programme also includes exercises for abdominal muscles, including pelvic floor exercises, to minimise the risk of developing stoma hernia [35]. However, to avoid heavy strain on the surgery wounds, restrictions regarding abdominal muscles are followed 6 weeks postoperatively. The exercise programme has been approved by the responsible medical surgeons. In addition to the structured exercise sessions, the patients are advised to take daily walks in their neighborhood. The number of recommended steps per day is set together with the physiotherapist based on the patient’s capability. To support the patient individual goal-setting, feedback and self-monitoring of daily steps are used similarly to those of the Activity Board. At the end of the exercise period, the physiotherapist recommends continued physical activity according to their clinical routines.

#### Control group

The control group will receive oral and written information of a gradually increased home-based exercise programme that includes daily walks and sit-to-stand exercises. They will also receive information on supportive techniques to improve physical activity, such as an activity diary, pedometer or a phone application.

### Outcomes

The measurements will be conducted using validated instruments at discharge (i.e. baseline), post-intervention (4 months) and 1 year after surgery (Table 1). With the purpose to receive information on the patients’ health and physical function before surgery (e.g., to adjust for in the analysis), measurements will also be conducted before surgery. All measurements will be conducted by experienced physiotherapist blinded to the intervention. A protocol for the measurements has been developed and the physiotherapist will receive specialised training by the research staff.

**Table 1:** Outcome measures and test occasions

Variable	Measure	Pre-op testing	Baseline	Post-testing	1-year follow-up
Physical function	6-min walk test	x	x	x	x
Gait speed	10-m walk test	x	x	x	x
Leg strength	Chair stand test	x	x	x	x
Handgrip strength	Jamar hand dynamometer	x	x	x	x
Habitual physical activity	ActivPAL3 micro		x	x	
Previous physical activity level^a^	Stanford Brief Activity survey	x			
Health-related quality of life	EORTC QLQ-C30 EORTC QLQ-BLM30	x	x	x	x
Fatigue	Piper fatigue scale	x	x	x	x
Psychological wellbeing	HADS	x	x	x	x
Pain	NRS	x	x	x	x
Length of hospital stay (days)	Medical records		x		
Complications	Medical records			x	x
Readmissions	Medical records			x	x

^a^Previous physical activity level is only used for adjustment purposes in the analysis

#### Primary outcome

Physical function, the primary outcome, will be measured using the validated Six-minute walk test (6MWT) [36, 37]. The test reproduces activity of daily living at a submaximal level, which is particularly applicable to elderly patients [33]. The patients are asked to walk as far as possible for 6 min. The number of meters (m), oxygen saturation and heart rate measured with a pulse oximeter will be recorded at the end of the test according to standard procedures [32]. The primary outcome variable will be walking distance in meters.

#### Secondary outcomes

Gait speed will be assessed with the 10-m walk test [37]. The test is used to determine walking speed in meters per second (m/s) over a short distance. The test is performed as three 10-m walks without assistance, one test walk, one in preferred walking speed and one in the fastest speed possible. Time is measured for the intermediate 6 m to allow for acceleration and deceleration. The outcome variable is m/s.

Grip strength will be assessed with the validated Jamar hydraulic hand dynamometer [38]. The patients will sit in a chair and hold the dynamometer. The test is performed three times for each hand and a mean value for each hand is calculated. The outcome variable is the grip strength reading in kg.

Leg strength will be assessed with the 30-s (sec) chair stand test [39]. The patient is asked to rise from a chair as many times as possible for 30 s. The outcome variable is the number of sit-to-stand transitions.

Habitual physical activity will be measured using the activPAL3 micro activity monitor (PAL Technologies Ltd., Glasgow, UK) [40, 41]. The ActivPAL is a small device which, when attached to the thigh, provides information based on position and acceleration of the body. The information is then transferred to body posture, the transition between postures, stepping and stepping speed. The activity monitor is attached to the anterior, midline of the thigh with dressing and does not provide feedback to the patient. The monitor will be worn for seven consecutive days after discharge from hospital and after the intervention period. Outcome variables will be 1) time spent sitting/lying, standing, stepping, 2) numbers of step counts and 3) sit-to-stand transitions.

Self-reported previous physical activity level will be measured using the 2-item Stanford Brief Activity Survey (SBAS) [42]. The SBAS assesses the usual amount and intensity of physical activity during the past year that a person performs. The first item describes five patterns of work activity, ranging from mostly sedentary to hard physical labour. The second item describes five patterns of leisure-time physical activity ranging from sedentary to regular vigorous-intensity aerobic activities. For respondents who are retired and have no job or regular work, they would select the response “not applicable”. For both items, the outcome variable was categorical and rated on a 5-point scale.

HRQoL will be assessed using the EORTC QLQ-C30 with addition of the EORTC QLQ-BLM30 questionnaire [43]. The EORTC QLQ-BLM30 is explicitly developed for patients with muscle-invasive urinary bladder cancer [44]. Scoring ranges from 0 (the worst) to 100 (the best) for functional health status and from 0 (the best) to 100 (the worst) for symptoms. Outcome variables will range from 0 to 100 for different domains.

Fatigue will be assessed using the Piper Fatigue Scale [45]. The questionnaire consists of 22 items and scoring ranges from 0 (none) to 10 (severe). The score is presented in four domains plus a total fatigue score. Outcome variables will range from 0 to 10.

Psychological wellbeing will be assessed using the Hospital Anxiety and Depression Scale (HADS) [46]. The scale consists of 14 questions, with each scored on a scale from 0 to 3, where 3 represents more symptoms. The questions are equally divided into the domains anxiety or depression; each domain can result in a maximum score of 21. Outcome variables will range from 0 to 21.

Pain will be assessed using the Numeric Rating Scale (NRS) [47]. The NRS is an eleven point scale and scoring ranges from 0 (no pain) to 10 (worst pain). The NRS is verbally delivered.

Data on length of stay at the hospital and frequency of readmission to hospital due to complications will be extracted from patient medical records. Readmissions will be extracted as 30 and 90 days after surgery and complications will be registered using the Clavien-Dindo classification [48, 49].

### Ethics

The project is approved by the Regional board of ethics in Stockholm (Dnr 2012/2214–31/4) and the Swedish Ethical Review Authority (Dnr 2020–01356).

The new model for rehabilitation is compared with similar care the patients are given in today’s care delivery. Yet, the control group will receive less attention than the intervention group we find it unethical to ask the patients to come to PHC, pay their visit and only receive, for example, stretching exercises. In addition, it will be challenging to motivate the physiotherapists in PHC to offer such treatment.

### Sample size

The primary outcome is physical function, evaluated with the validated 6MWT. Based on data from our pilot study [28], we calculate an increase in 100 m in the intervention group, 70 m in the control group and a standard deviation of 30 m. To obtain a statistical power of 80% with a type 1 error set at 0.05 32 patients are needed (16 in each group). However, as the test is highly correlated with sex and age [37] we will stratify the analysis and increase the sample size.

For the secondary outcome readmissions due to complications, we will estimate 20% readmissions in the intervention group compared with the proportion of patients being readmitted today, which is 45% in the control group. To obtain a statistical power of 80% with a type 1 error of 0.05, 112 patients are needed (56 in each group). Taken all this into account and guard against dropout, 120 patients (60 in each group) will be included in the study.

### Data management and study database

Data will be entered using an electronic system (PheedIt), which is based on the SAS system provided and operated by the CTO at the Centre for Clinical Cancer studies, Theme Cancer, Karolinska University Hospital, Solna, Sweden. A data management plan is delivered by CTO, documenting the database and all procedures for data management. The investigator verifies that all data entries in the case report forms (CRFs) are accurate and correct. If certain assessments according to the protocol are not performed for any reason, or if certain information is not available, not applicable or unknown, this will be indicated in the CRF by the investigator. The investigator is required to sign off all reported data.

### Source data

In this study physical tests, movement sensor data, patient-reported outcome measures and medical records will be regarded as source data.

### Coding

In the study database patients will be identified only through the unique randomisation number. All data will be stored with coded identification and no access to the patients’ ID. Patient identification will not be revealed in text files. Logbooks with identification numbers and the respective codes will be stored in a locked environment at the local centre that is not accessible to personnel not involved in conducting the trial.

### Statistical analysis

Descriptive statistics will be performed to ensure comparability between data at baseline. To evaluate the effect of the intervention mixed models or linear regression models (SPSS Inc., Chicago, IL, USA) according to the intention to treat procedure and with an alpha level of 0.05 will be used. Significance of main or interaction effects will be explored using the Bonferroni posthoc multiple comparison test. In the case of skewed distribution logarithmic transformations or corresponding nonparametric statistics will be used to assess the effect of the intervention.

### Implementation process

Because the intervention design is intricate, we will, in addition to testing effects of the CanMoRe programme on patient-level outcome measures, also observe and gather information on factors that might have influenced the implementation of the programme [50]. The evaluation of the implementation process will be based on the Medical Research Council guide for process evaluation of complex interventions [51]. The knowledge gained can also be used to offer recommendations on which strategies to use when implementing the CanMoRe programme in other clinical settings and on a large scale.

The initial strategies for the process evaluation will include 1) meetings with surgeons, the head of the surgical ward and PHC clinics, 2) discussions and involvement with physiotherapists and nurses at the surgical ward and physiotherapists at PHC clinics and 3) education of the CanMoRe programme and outcome measures to physiotherapists who will be involved in the intervention. A leaflet for the patients, an extended educational leaflet and a short education for the physiotherapists have been developed.

To study what is delivered measures of fidelity, dose, adaptation and reach will be assessed. Fidelity relative to the CanMoRe programme will be evaluated as the extent to which the programme was delivered as expected. Dose will be assessed as the quantity of the intervention (the CanMoRe programme and the education of physiotherapists in PHC) implemented. Adaptation, such as changes done to fit different PHC settings, will be reported in a questionnaire. Reach will be assessed regarding how many eligible patients signed an informed consent form and how many in the intervention group fulfilled the CanMoRe programme. In addition, adverse events will be registered.

Context includes external factors that may act as a barrier or facilitator to both implementation itself and the patient level effect. Assessing barriers and facilitators to programme implementation will also involve evaluating programme feasibility, i.e. the extent to which patients and health care staff regard the CanMoRe as satisfactory in terms of content and complexity/difficulty. We plan to conduct an interview study on patients’ experiences of the intervention. In addition, we plan to collect information on possible barriers that might have influenced the implementation of the programme and facilitators that might have supported it at the various clinical sites. Both qualitative (structured observation, focus groups and individual semi-structured interviews) and quantitative (questionnaires and enrolment files) methods will be used to assess how the intervention was delivered as well as experiences of the different stakeholders (patients, physiotherapists and other health care staff as well as managers).

By using several sources for data collection, triangulation can be achieved, which supports the trustworthiness of the study. The Consolidated Framework for Implementation Research (CFIR) will be used in the current study to guide the investigation of context, i.e. potential barriers and facilitators of the implementation process. Constructs that we believe specifically impact the implementation outcomes in the present study and guidelines for interview questions and observation protocols published by CIFR will be followed (http://cfirguide.org/tools.html).

## Discussion

To our knowledge, this is the first study to examine the effects of individually targeted exercise in PHC compared with traditional advice on home exercise training after RARC. Moreover, the study will include a process evaluation of factors thought to influence the implementation of the programme.

Although there is evidence that exercise is beneficial to improve physical function, physical activity, HRQoL, reduce fatigue and perhaps reduce complications, it is essential to design feasible and easy to implement interventions in a health care setting. Our intervention is individually targeted [34] and designed based on current global guidelines for physical activity and exercise [12], strategies for behavioural support [17] and adapted to fit within the PHC structure. The length of the intervention is based on exercise principles [52] and what is feasible to conduct within PHC. Yet, this study does not tell us whether the length of the intervention is the optimal length for best health benefits, nor if a booster session after the intervention period is needed. The dose of exercise has been previously tested in a pilot study and showed a positive effect on physical function, was safe and had no adverse events [28]. After the pilot study, we revised the exercise programme to be individually based but still include aerobic and muscle-strengthening exercises. The addition of behaviour support to our programme (e.g., goal setting, graded tasks, self-monitoring and feedback) has been shown to be associated with increased physical activity behaviour [53].

At discharge from the hospital, the standard care for patients includes information on the importance of physical activity. In this study the control group receive a light intervention, i.e. recommended daily walks and leg-strengthening exercises instead of the standard care. The exercise recommendation is low dose and not specific but can still affect the results by producing a smaller difference between the groups. Because there is strong evidence for the effect of physical activity, we find it unethical not to give the control group any advice on physical activity. At discharge, many patients are feeble because of surgery and the postoperative period at hospital, which can also result in fear of movement. These patients require supervised physical exercise, as in the intervention group in this study.

The process evaluation using measures of fidelity, dose, adaption, adherence and reach as well as patient perspectives and experiences of the programme can help explain the results. In addition, it can speed up the process of translating findings from research settings to clinically representative settings. The programme fits well within the healthcare system and the exercises are generic to those recommended for cancer survivors and the tools for motivational support are generic for the whole population. If the programme is proven effective, it should be generalisable to other patient groups.

There are some limitations. First, due to the difficulties of double-blinding, we have a single-blind design in which a physiotherapist that is not involved in the programme is conducting the measurements. Second, we foresee a long recruitment period that can lead to a change in health care staff/physiotherapists in PHC. To ensure quality we plan to have continued contact with the clinics and a new educational structure for training if needed.

In summary, this proposed RCT has the potential to provide new knowledge within rehabilitation after radical cystectomy for urinary bladder cancer. The programme should be readily applied to other patient groups undergoing abdominal surgery for cancer and has the potential to change the health care chain for these patients.

## Supplementary information

**Additional file 1.** Exercise programme.

## Data Availability

The datasets generated during and/or analysed during the current study are not publicly available due to Swedish and EU personal data legislation but are available from the corresponding author on reasonable request. Any sharing of data will be regulated via a data transfer and user agreement with the recipient.

## Abbreviations

CanMoRe: Cancer mobilisation rehabilitation
CFIR: Consolidated framework for implementation research
CRF: Case report form
CTO: Clinical trials office
HADS: Hospital anxiety depression scale
HRQoL: Health-related quality of life
MCT: Movement continuum theory
m/s: Meters per second
PHC: Primary health care
QoL: Quality of life
RARC: Robotic-assisted radical cystectomy
RCT: Randomised controlled trial
RR: Responsible researcher
SBAS: Stanford brief activity survey
sec: Seconds
SPIRIT: Standard protocol items: recommendations for interventional trials
6MWT: Six-minute walk test
