# Supplementary material for: The CanMoRe trial – evaluating the effects of an exercise intervention after robotic-assisted radical cystectomy for urinary bladder cancer: the study protocol of a randomised controlled trial
Source: BMC Cancer. 2020 Aug 26;20:805. doi: 10.1186/s12885-020-07140-5 (PMC7448437; doi:10.1186/s12885-020-07140-5)
Supplement: Supplementary file 1 — Additional file 1. Exercise programme. [file 12885_2020_7140_MOESM1_ESM.zip › 12885_2020_7140_MOESM1_ESM/Additional file 1_Exercise program CanMoRe English translationR0.pdf]

Translation of Swedish origin to English

Exercise Program CanMoRe study

Occasion 1

• Short anamnesis

• Instruction of exercise program for pelvic floor and abdominal muscles (See next page). This should then be performed daily as a home exercise and followed up after 2 weeks

• Demonstration of the gym before the next training session

Occasion 2 onwards

Warm up

• 5 minutes

• Rehab bicycle (large saddle) or walking belt or cross trainer

Strength Training 1: Training Week 1 and 2

Light load: 2 x 15 reps, ie about 50-70% of 1RM

• Squat with big ball between back and wall

• Lats pull with Theraband

• Theraband, chest

• Push-ups against wall

• heel raises

Strength training 2: from training week 3

Increased load: 2 x 10 reps, ie about 65 - 75% of 1RM

• Leg press, sequencer

• Lats / Pulldown, Sequencer

• Rowing, sequencer

• Chest press, sequencer

• heel raises

Fitness training: intervals

• Rehab bike (big saddle) or cross trainer

• Effort is estimated using BORG's RPE scale.

• Training Week 1-5:

o 3 5 - 10 minute intervals

o Moderate intensity 40-59% VO2max, BORG 12-13

Translation of Swedish origin to English

o 2-5 minute rest between intervals

• Training week 6-12:

o 3 5 - 10 minute intervals

o Moderate-high intensity, 40-80% VO2max, BORG 12-15

o 2-5 minute rest between intervals

Flexibility training: individually as needed

2020-06-05

Hanna Johansson
