# Supplementary material for: The CanMoRe trial – evaluating the effects of an exercise intervention after robotic-assisted radical cystectomy for urinary bladder cancer: the study protocol of a randomised controlled trial
Source: BMC Cancer. 2020 Aug 26;20:805. doi: 10.1186/s12885-020-07140-5 (PMC7448437; doi:10.1186/s12885-020-07140-5)
Supplement: Supplementary file 1 — Additional file 1. Exercise programme. [file 12885_2020_7140_MOESM1_ESM.zip › 12885_2020_7140_MOESM1_ESM/Additional file 1_Exericse program_sweR0.pdf]

Träningsprogram

Tillfälle 1

- Kort anamnes
- Instruktion av träningsprogram för bäckenbotten och magmuskler (Se nästa sida). Detta ska sedan utföras dagligen som hemträning och följas upp efter 2 veckor
- Demonstration av gymmet inför nästa träningstillfälle

Tillfälle 2 och framåt

Uppvärmning

- 5 minuter
- Rehabcykel (stor sadel) alternativt gåband eller crosstrainer

Styrketräning 1: *träningsvecka 1 och 2*

Lätt belastning: 2 x 15 reps, det vill säga ca 50-70 % av 1RM

- Knäböj med stor boll mellan rygg och vägg
- Latsdrag med Theraband
- Theraband, bröstrygg
- Armhävning mot vägg
- Tåhävningar

Styrketräning 2: *från träningsvecka 3*

Ökad belastning: 2 x 10 reps, det vill säga ca 65 – 75 % av 1RM

- Benpress, sekvensapparat
- Latsdrag/Pulldown, sekvensapparat
- Rodd, sekvensapparat
- Bröstpress, sekvensapparat
- Tåhävningar

Konditionsträning: *intervaller*

- Rehabcykel (stor sadel) eller crosstrainer
- Ansträngning skattas med BORGs RPE-skala.
- Träningsvecka 1-5:
    - 3 stycken 5 - 10 min intervaller
    - måttlig intensitet 40-59 % VO2max, BORG 12-13
    - 2-5 minuters vila mellan intervaller
- Träningsvecka 6-12:
    - 3 stycken 5 - 10 min intervaller
    - måttlig-hög intensitet, 40-80 % VO2max, BORG 12-15
- 2-5 minuters vila mellan intervaller

Rörelseträning: *individuellt efter behov*

KAROLINSKA UNIVERSITY HOSPITAL

Karolinska Institutet

Träningsprogram för bäckenbotten- och magmuskler

Vi rekommenderar att du gör detta program vid ett tillfälle per dag.

Du som har ett ortotopt blåssubstitut gör de bäckenbottenövningar **A, B, C** du fått information om av uroterapeuten fyra gånger per dag, och vid ett tillfälle per dag lägger du till övning **D, E, F**.

Bäckenbotten som består av muskulatur och fibrös vävnad, utgör ”golvet” i bukhålan. Den sträcker sig från blygdbenet till svanskotan och fäster även på sittbensknölarna. Muskulaturens funktion är bland annat att reglera avföring, gas- och urinavgång. Bäckenbotten består av små tunna muskler. En sammandragning av bäckenbottenmuskulaturen är därför aldrig stor och kraftig.

Om du har kateter i urinröret, avvakta med magträning till katetern är borttagen.

Ligg bekvämt på rygg. Slappna av och ta fem djupa lugna andetag innan du börjar med träningsprogrammet.

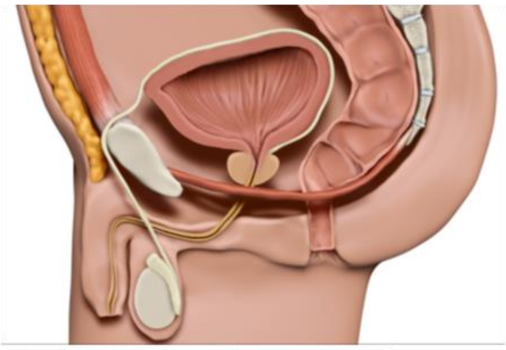

***Bild** från Karolinska Universitetssjukhuset*

Bäckenbotten omsluter ändtarmsöppningen och urinröret

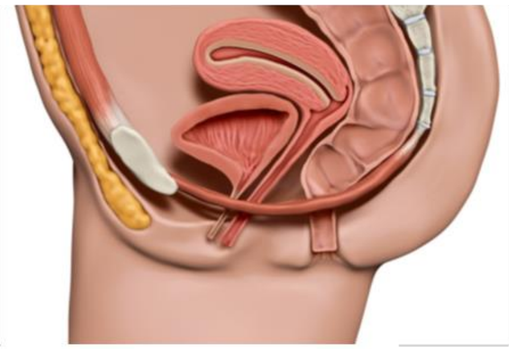

***Bild** från Karolinska Universitetssjukhuset*

Bäckenbotten omsluter ändtarmsöppningen, slidan och urinröret

A ”Hitta rätt” /uppvärmningsknip

1 Ligg bekvämt t.ex. på rygg med böjda ben, gärna med en kudde under knäna.

2 Knip med musklerna som om du vill hindra gasavgång eller som att hålla dig då du är kissnödig.

3 Knip med lätt till måttlig kraft i 3 sekunder - vila i 3 sekunder.

4 Upprepa övningen 10-15 gånger.

B Styrkeknip

1 Knip på samma sätt som i övningen ovan, men knip nu **så hårt** du kan.

2 Håll knipet i 5-6 sekunder - slappna av i 10 sekunder.

3 Upprepa övningen 5-10 gånger.

C Uthållighetsknip

1 Knip fast och bestämt, **så länge du kan.**

2 Utför övningen 1 gång per träningstillfälle

När du är säker på att du bara aktiverar bäckenbotten och ingen annan muskelgrupp kan du naturligtvis göra övningarna sittande eller stående.

KAROLINSKA UNIVERSITY HOSPITAL

Karolinska Institutet

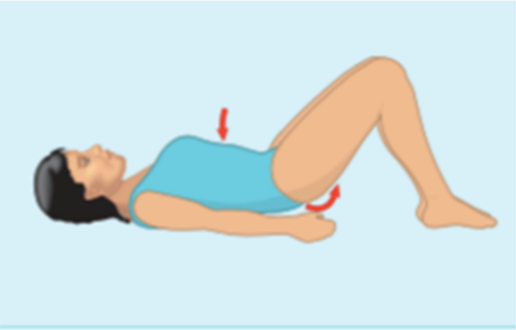

D Bäckentippning
1 Ligg på rygg på en plan yta, med böjda ben och fotsulorna mot underlaget.
2 Dra in magen, tippa stjärten lite uppåt samtidigt som du pressar svanken ned mot underlaget, håll i 2 sekunder.
3 Slappna av långsamt
4 Upprepa 10 gånger

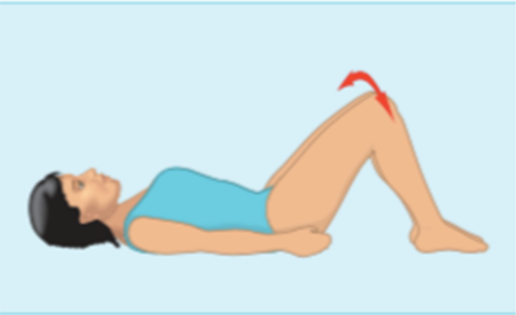

E Knärullning
1 Ligg på rygg på en plan yta, med böjda ben och fotsulorna mot underlaget.
2 Dra in magen, håll ihop knäna, fäll knäna långsamt från sida till sida.
3 Upprepa 10 gånger

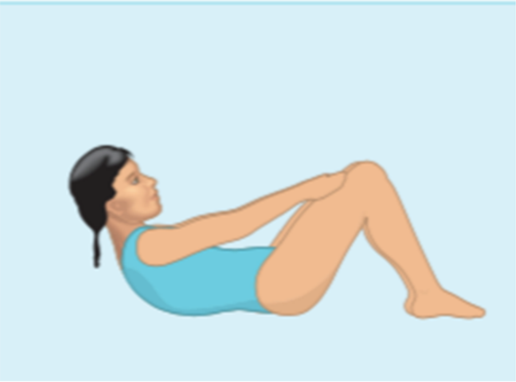

***Bild** från Thompson, 2007*

F Anpassad Sit ups
1 Ligg på rygg på en plan yta, med böjda ben och fotsulorna mot underlaget.
2 Placera händerna på lårens framsidor, dra in magen.
3 Lyft huvudet från kudden
4 Håll i 3 sekunder, gå sedan långsamt tillbaka till utgångsposition.
5 Upprepa 10 gånger

Ansvariga för genomförandet av studien:

Andrea Porserud
Specialistsjukgymnast
FO Arbetsterapi och Fysioterapi
Karolinska Universitetssjukhuset
Doktorand
Karolinska Institutet
Tel: 08 - 517 725 28

Patrik Karlsson
Leg Fysioterapeut
FO Arbetsterapi och Fysioterapi
Karolinska Universitetssjukhuset
Doktorand
Karolinska Institutet
08 – 517 725 28

Maria Hagströmer
Professor, leg sjukgymnast
Sophiahemmets Högskola
Karolinska Institutet

Markus Aly
Urolog, Tema Cancer
Karolinska Universitetssjukhuset
Med dr, Karolinska Institutet
